# Trap Polarity and the *p*/*n* Asymmetry in Oxidised DNTT: A Frontier-Shift Rule

**DOI:** 10.3390/ma19153333

**Published:** 2026-08-05

**Authors:** Matej Matuš, Tomáš Vincze, Michal Hanic, Lubica Stuchlikova, Martin Weis

**Affiliations:** Institute of Electronics and Photonics, Faculty of Electrical Engineering and Information Technology, Slovak University of Technology in Bratislava, Ilkovičova 3, 841 04 Bratislava, Slovakia; matej.matus@stuba.sk (M.M.); tomas.vincze@stuba.sk (T.V.); michal.hanic@stuba.sk (M.H.); lubica.stuchlikova@stuba.sk (L.S.)

**Keywords:** DNTT, organic thin-film transistor, charge traps, atmospheric oxidation, hydroxyl defect, GFN2-xTB, DLTFS, frontier orbitals, ambient stability

## Abstract

Organic thin-film transistors based on dinaphtho[2,3-*b*:2′,3′-*f*]thieno[3,2-*b*]thiophene (DNTT) are attractive for low-cost, large-area electronics, but in unencapsulated devices, atmospheric oxidation generates charge traps whose electronic character—which product traps holes and which traps electrons—has not been mapped systematically. Here, 39 oxygen- and hydroxyl-related defect identities of DNTT are screened with the semi-empirical GFN2-xTB method, complemented by an a priori frontier reactivity index, and classified by the sign of the frontier-level shift. This sign obeys a simple rule: a net π-donating hydroxyl raises the HOMO and yields a hole trap, whereas a net π-accepting carbonyl or quinone lowers the frontier levels and yields a deep electron trap. Hybrid density-functional theory (B3LYP/def2-TZVP) confirms the sign rule and the ordering of the shifts across all closed-shell defect classes. The rule provides a compact, defect-level rationalisation of the well-known asymmetry whereby *p*-type acenes tolerate air far better than *n*-type ones. Finally, a hole trap of about 0.255 eV, measured by deep-level transient Fourier spectroscopy, is shown to be consistent with a hydroxyl-related origin, without claiming a unique microscopic assignment.

## 1. Introduction

Organic semiconductors have been envisioned for a wide range of low-cost, flexible and large-area electronic applications, including displays, light-emitting diodes, photovoltaics, and sensors [1,2,3,4]. Their performance is, however, commonly governed by charge traps—that is, localised states within the transport gap that capture and re-emit carriers—so that a deep understanding of the origin and of the electronic character of these traps is crucial for both device operation and long-term stability [5].

Among the small-molecule semiconductors, DNTT has become a benchmark *p*-type material, owing to its high hole mobility and its comparatively good ambient stability [6]. Even so, in unencapsulated devices, the semiconductor is inevitably exposed to oxygen and humidity, and its electrical characteristics gradually drift [7,8]. This degradation has usually been attributed to physical causes—water-related trapping at the semiconductor/dielectric interface [7,9] or morphological reorganisation and grain-boundary disorder [8,10]—and the microscopic identity of the trapping species, in particular whether a given species traps holes or electrons, has commonly remained ambiguous. A chemical modification of the semiconductor itself, and the electronic signature it would leave, has by contrast received comparatively little attention.

Two complementary lines of investigation have commonly been pursued. On the experimental side, deep-level transient spectroscopy and its Fourier variant (DLTS/DLTFS) provide the activation energy and the capture cross-section of individual trap levels [11,12,13], yet they report an energy rather than a chemical structure. On the theoretical side, quantum-chemical calculations can associate a candidate defect with a frontier-level shift [14,15], while the photo-oxidation chemistry of acenes—proceeding through endoperoxides and quinones—has been studied in its own right [16,17,18]. The difference between these approaches lies in what they deliver: a measured energy on the one hand, and a molecular structure on the other.

What is still missing is a systematic bridge between the two, i.e., a map that links the chemical identity of an atmospheric oxidation product to the electronic character—and, above all, the polarity—of the trap that it produces. Furthermore, the well-known experimental asymmetry, whereby *p*-type acene devices survive air far better than their *n*-type counterparts [7,8], has usually been attributed to oxidation in rather general terms, but it has not been rationalised at the level of individual defect states.

In this study, we address these gaps by screening a broad family of 39 oxygen- and hydroxyl-related defect identities of DNTT with the semi-empirical GFN2-xTB method [19], complemented by an a priori frontier reactivity index and cross-checked against hybrid density-functional theory. We show that the sign of the frontier-level shift governs the trap polarity, which in turn rationalises the *p*/*n* ambient-stability asymmetry of acene semiconductors.

## 2. Materials and Methods

**Figure 1:**
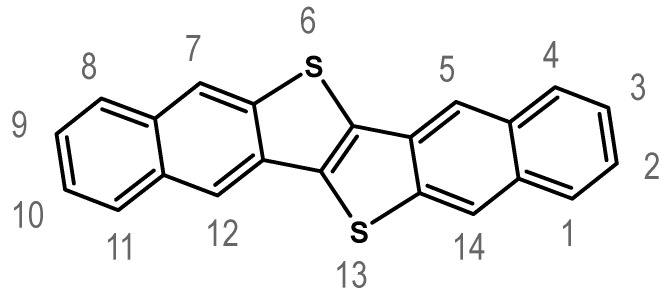
Molecular structure of dinaphtho[2,3-*b*:2′,3′-*f*]thieno[3,2-*b*]thiophene (DNTT) with the IUPAC atom numbering used throughout this work; the two sulfur atoms occupy positions 6 and 13. All defect locants in the text refer to this numbering. DNTT belongs to the $C_{2h}$ point group (with an inversion centre at the molecular centroid), so the ring C–H positions occur in symmetry-equivalent pairs and only six distinct C–H environments exist; these six symmetry-unique sites are the ones ranked by the frontier reactivity index.

The molecular structure of DNTT, together with the IUPAC atom numbering used throughout this work, is shown in Figure 1.

### 2.1. Device Fabrication

The DNTT thin-film transistors were fabricated in a bottom-gate, top-contact configuration on heavily doped silicon wafers, on which a thermally grown SiO_2_ layer (100 nm) served as the gate insulator. Prior to processing, the substrates were cleaned in successive ultrasonic baths of a 20 wt% aqueous monoethanolamine solution, isopropyl alcohol and acetone (15 min each), and were subsequently exposed to oxygen plasma in order to remove residual organics and to modify the surface wettability. The cleaned substrates were then immersed for 1 h in a 1 mM solution of octadecyltrichlorosilane (OTS, >99%) in toluene, followed by annealing at 125 °C for 10 min. The DNTT (99%) active layer was grown on the OTS-modified surface by physical vapour deposition (Spectros 100, Kurt J. Lesker, Hastings, UK) at a pressure below $10^{-6}$ Pa through shadow masks, at a rate of 3 nm s^−1^ monitored by a quartz crystal microbalance, giving a semiconductor thickness of 50 nm. Silver electrodes (80 nm, 99.99%) were then deposited through a shadow mask without breaking the vacuum. All chemicals were supplied by Merck (Darmstadt, Germany) and used as received.

### 2.2. DLTFS Measurement

The transistors were left unencapsulated. They were first measured approximately one day after fabrication, and all measurements were completed within one week; for most of this period, the samples were held under vacuum in the cryostat so their cumulative ambient exposure was short. The channel width was W=2.5 mm, while the channel length was varied from 50 to 200 μm; the extracted trap parameters were found to be essentially independent of *L*, the only appreciable difference in the total capacitance (see the C–V characteristics in the Supplementary Materials). The *p*-type operation was confirmed by hole accumulation at negative gate bias in the capacitance–voltage (C–V) characteristic. Deep-level transient Fourier spectroscopy (DLTFS) was carried out on a DL8000 system (BIO-RAD Micromeasurement, Mountain View, CA, USA), employing optical excitation (a green LED, λ=520 nm) rather than an electrical filling pulse to populate the traps at a constant applied bias. Measurements were carried out over the temperature range 130–205 K, with the bias $U_{R}$ varied from −15 to +10 V, an optical filling-pulse duration $t_{p}$ between 0.1 and 0.5 s, and a period width $T_{W}$ between 1 ms and 10 s. The hole trap analysed in Section 3.5 was resolved at $U_{R}=-10$ V, $t_{p}=100$ ms and $T_{W}=1$ s, and its activation energy was extracted from an Arrhenius analysis of the thermal emission time constant $\tau_{e}$.

### 2.3. Computational Screening

Candidate atmospheric oxidation defects were screened with the semi-empirical tight-binding method GFN2-xTB [19]. For each defect, an input structure was generated with an RDKit-validated script [20] using standard bond lengths (C–O_phenolic_ 1.36 Å, C=O_carbonyl_ 1.22 Å, O–O 1.47 Å, O–H 0.96 Å), the geometry was optimised in the gas phase to a gradient norm below $10^{-3}$ E_h_/a_0_, and a single-point calculation gave the frontier-orbital energies, Mulliken charges, and dipole moment. Closed-shell singlet states (uhf=0) were used for hydroxyls, hydroperoxides, quinones, and cyclic defects; open-shell doublets (uhf=1) were used for peroxyl, alkoxyl, and carbonyl-radical intermediates. In total, 39 electronic identities across six chemical classes were mapped. For pristine DNTT this protocol gives HOMO =−9.7598 eV, LUMO =−7.7751 eV, and a HOMO–LUMO gap of 1.985 eV, which is used throughout as the reference.

To test the robustness of the frontier-level shifts against a higher level of theory, a subset of fourteen representative closed-shell defects was recomputed with density-functional theory. Single-point B3LYP-D3(BJ)/def2-TZVP calculations [21], accelerated by the RIJCOSX approximation with the def2/J auxiliary basis, were carried out on the GFN2-xTB geometries with ORCA 6.1.1 [22]; the def2-SVP results were additionally checked for basis-set consistency. The comparison with the semi-empirical shifts is presented in Section 4.4.

### 2.4. Frontier Reactivity Index

To rank the intrinsic susceptibility of each C–H position to oxidative attack a priori—i.e., independently of the trap energetics—the Mulliken HOMO population of pristine DNTT was computed at the HF/STO-3G level with PySCF [23]. This frontier population is used only as an ordinal reactivity index; because a minimal basis and Mulliken partitioning are known to be basis-set dependent, no quantitative weight is placed on its absolute values. The peri positions (5 and 12) carry the largest HOMO population and are therefore the most reactive site.

### 2.5. Choice of the Defect Set

**Figure 2:**
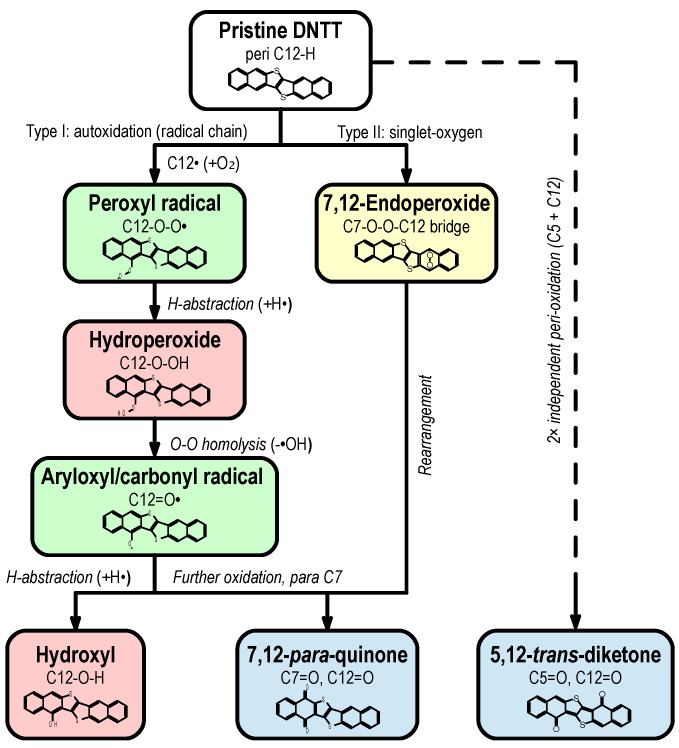
Rationale for the computed defect set: The two established photo-oxidation channels of acenes at the peri position of DNTT. Type I (radical-chain autoxidation) proceeds through a peroxyl radical, a hydroperoxide and an aryloxyl/carbonyl radical; the last branches to a stable hydroxyl (hole trap) or, on further oxidation at the *para* carbon, to the 7,12-*para*-quinone (electron trap). Type II (singlet-oxygen) forms the 7,12-endoperoxide, which rearranges to the same quinone. Both channels converge on the 7,12-*para*-quinone; the 5,12-diketone (dashed path, formed by two independent oxidations) is a distinct species. Colours denote the electronic character (hole trap, electron trap, gap-widener, transient open-shell radical); the open-shell (SOMO) levels are not directly comparable with the closed-shell HOMO/LUMO and are quantified separately below.

The set of computed defects was chosen to represent both established channels of acene photo-oxidation rather than to sample chemical space at random, the peri position—which the frontier reactivity index identifies as the most reactive site—being taken as the representative point of attack. In the Type I (radical-chain autoxidation) channel, molecular oxygen adds to a carbon radical to give a peroxyl radical, which abstracts hydrogen to form a hydroperoxide; O–O homolysis then yields an aryloxyl/carbonyl radical, from which the sequence branches either to a stable hydroxyl or, upon further oxidation at the *para* carbon, to a *para*-quinone. In the Type II (singlet-oxygen) channel, the same ring forms an endoperoxide, which rearranges to the corresponding *para*-quinone. Both channels therefore converge on the 7,12-*para*-quinone as the common, stable end-product (Figure 2), whereas the 5,12-diketone—formed instead by two independent oxidations across the molecule—is retained as a distinct electronic species. It should be noted here that the radical intermediates are kept only to complete the mechanistic picture: being open-shell, their singly occupied (SOMO) levels are treated separately and are not placed on the same quantitative axis as the closed-shell products.

### 2.6. From Molecular Orbitals to Trap Levels

Following the common picture of an isolated defect molecule embedded in the pristine matrix [5], we quantify each defect by the shift of its frontier levels relative to pristine DNTT,

(1) $$\Delta E_{\mathrm{HOMO}}=E_{\mathrm{HOMO}}^{\mathrm{defect}}-E_{\mathrm{HOMO}}^{\mathrm{pristine}},\;\Delta E_{\mathrm{LUMO}}=E_{\mathrm{LUMO}}^{\mathrm{defect}}-E_{\mathrm{LUMO}}^{\mathrm{pristine}}.$$

With the sign convention used here, a positive $\Delta E_{\mathrm{HOMO}}$ places a localised, donor-like state above the valence-band maximum (VBM) of the matrix (a hole trap), whereas a negative $\Delta E_{\mathrm{LUMO}}$ places a state below the conduction-band minimum (an electron trap).

Two limitations of this proxy are stated up front and shape every claim below. First, the calculations are single-molecule and gas-phase, and GFN2-xTB is a semi-empirical method: its absolute orbital energies are not reliable (the pristine HOMO of −9.76 eV is far from the reported ionisation energy of DNTT of ≈−5.4 eV), and only relative shifts and their ordering are treated as meaningful. Accordingly, we report trends rather than absolute trap depths. Second, a localised charge polarises the surrounding molecular crystal, stabilising the trap by a polaronic term $P^{+}\approx 0.05$–0.15 eV for acene-like matrices ($\varepsilon_{r}\approx 3.6$) [24,25,26]. Because this term is, to a good approximation, a state-independent rigid offset, it shifts the whole map downward without changing the sign of $\Delta E_{\mathrm{HOMO}/\mathrm{LUMO}}$ or the ordering of defects. We therefore do not compute an explicit crystal-polarisation cell and do not claim absolute trap depths; the conclusions drawn below—trap polarity and trends—are invariant to $P^{+}$ within its literature range.

## 3. Results

### 3.1. Frontier Reactivity of Pristine DNTT

**Figure 3:**
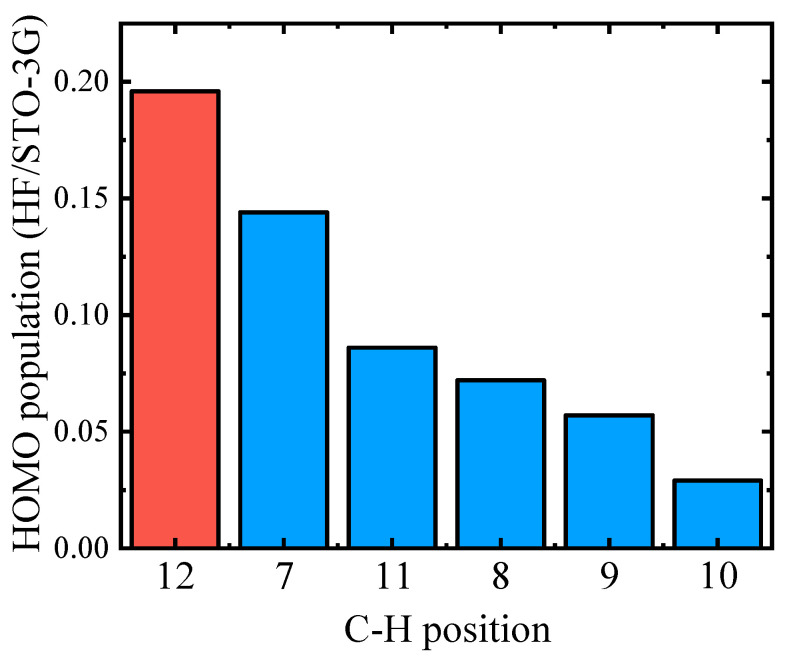
Frontier reactivity of pristine DNTT: the Mulliken HOMO population at each symmetry-unique C–H position (HF/STO-3G), used as an ordinal index of susceptibility to photo-oxidative attack. The population is largest at the peri position (12) and decreases towards the meta-terminal position (10); the locant numbering follows Figure 1.

As a starting point, the intrinsic susceptibility of the aromatic skeleton to oxidative attack was mapped through the frontier (HOMO) electron density of pristine DNTT (Figure 3). The Mulliken HOMO population analysis resolves six symmetry-unique C–H positions with markedly different frontier densities: the population reaches a value of 0.196 at the peri position (12) and decreases progressively through 0.144 (7), 0.086 (11), 0.072 (8), and 0.057 (9) down to 0.029 at the meta-terminal position (10). Obviously, this gradient orders the sites by their expected reactivity and identifies the peri position as the one most susceptible to electrophilic—and hence photo-oxidative—attack.

It should be noted here that this frontier density is not merely a predictor of where oxidation occurs but also of how deep the resulting trap will be: for the mono-hydroxyls, the HOMO shift follows an approximately linear relation with the local HOMO population, $\Delta E_{\mathrm{HOMO}}\;\left(\mathrm{eV}\right)\approx 0.85\times {\mathrm{HOMO}}_{\mathrm{pop}}+0.015$. In other words, one and the same quantity governs both the site selectivity and the trap depth so that the most reactive peri site also yields the deepest hole trap of the mono-hydroxyl family (+0.181 eV). This single physical thread—the local frontier density—underlies the trends discussed in the following sections.

### 3.2. The Sign of the Frontier Shift Sets the Trap Polarity

The central and, at first sight, almost trivial organising principle of the map is that the sign of the frontier-level shift—and hence whether a given oxidation product behaves as a hole or as an electron trap—is governed by whether the oxygen functionalisation is, on balance, π-donating or π-accepting at the site in question.

**Figure 4:**
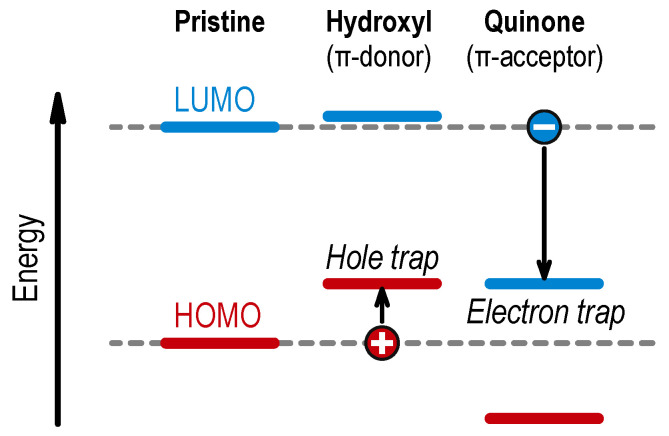
Sign rule for the frontier shift. A net π-donating substituent (phenolic –OH) raises the HOMO and produces a donor-like hole trap ($\Delta E_{\mathrm{HOMO}}>0$); a net π-accepting substituent (carbonyl/quinone C=O) lowers the HOMO and introduces a deep $\pi^{*}$ LUMO, producing an electron trap ($\Delta E_{\mathrm{LUMO}}<0$).

Hydroxylation is the archetypal π-donor: the phenolic oxygen lone pair is donated into the aromatic π system (i.e., the +M effect), thereby raising the HOMO. As a result, all six unique mono-hydroxyls yield $\Delta E_{\mathrm{HOMO}}>0$, that is, donor-like hole traps, and the shift is regulated by the local HOMO density, reaching a value of about +0.181 eV at the most reactive peri site. Oxidation to a carbonyl/quinone, on the other hand, is the archetypal π-acceptor: in the 7,12-quinone, the C=O groups withdraw π density and introduce a low-lying $\pi^{*}$ level so that the LUMO drops by $\Delta E_{\mathrm{LUMO}}=-1.289$ eV (i.e., a deep electron trap), while the HOMO simultaneously falls below the pristine VBM ($\Delta E_{\mathrm{HOMO}}=-0.506$ eV) and the defect is therefore not a hole trap. Hence, the two stable end-members of the oxidation sequence dwell on opposite sides of the trap-polarity divide, and, crucially, this divide follows directly from substituent electronics rather than from any fitted quantity (Figure 4).

### 3.3. Competition Between Donation and Withdrawal: The Hydroperoxide Cross-Over

That the sign is set by a competition—and not merely by the presence of oxygen—is illustrated most cleanly by the hydroperoxides (C–OOH), in which π-donation from the α-oxygen lone pair (+M) competes against σ-withdrawal along the electronegative O–O bond (−I). Interestingly, the outcome is governed by the local frontier density: at the peri site, where the HOMO density is the highest, donation prevails and the defect remains a hole trap ($\Delta E_{\mathrm{HOMO}}=+0.088$ eV), whereas at the low-density terminal positions, withdrawal prevails and $\Delta E_{\mathrm{HOMO}}$ becomes negative, reaching −0.144 eV at C10. In other words, one and the same chemical group produces opposite trap polarity, depending solely on where it is attached to the molecule—a clear demonstration that the trap polarity is governed by the product of the local frontier density and the substituent electronics.

The same trend is reflected in the charge on the α-oxygen, which tracks a monotonic loss of donor strength across the three single-oxygen species at the peri site (Table S3): hydroxyl q(αO)≈−0.38 ($\Delta E_{\mathrm{HOMO}}=+0.181$ eV), hydroperoxide ≈−0.17 (+0.088 eV), and peroxyl radical ≈−0.04 (+0.052 eV). It should be noted here that the hydroperoxide is thus not simply a “weaker hydroxyl” but rather a distinct electronic identity, the sign of which may invert along the molecule.

### 3.4. The Defect Landscape: Six Electronic Regimes

**Figure 5:**
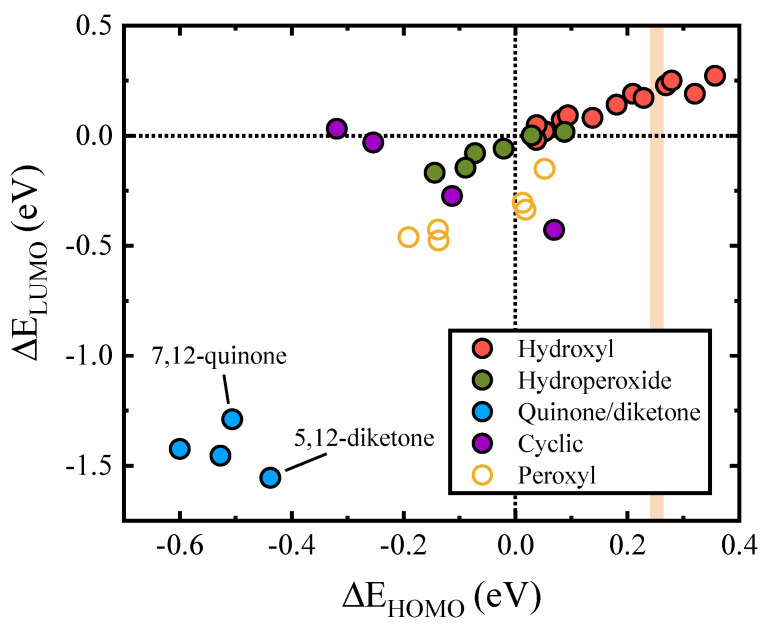
Frontier-shift landscape of the DNTT oxidation defects: the LUMO shift $\Delta E_{\mathrm{LUMO}}$ versus the HOMO shift $\Delta E_{\mathrm{HOMO}}$ (relative to pristine DNTT) for the 33 closed-shell and peroxyl species; the six alkoxyl/carbonyl radicals are singly occupied and are omitted (see Table S1). Colour denotes the chemical class, and filled versus open markers distinguish closed-shell from open-shell (peroxyl) species. The dashed lines mark the pristine VBM ($\Delta E_{\mathrm{HOMO}}=0$) and CBM ($\Delta E_{\mathrm{LUMO}}=0$), and the orange band indicates the measured hole trap at $\Delta E_{\mathrm{HOMO}}\approx 0.255$ eV (a proxy). Hydroxylation populates the shallow hole-trap region (right), whereas oxidation to the quinones and the diketone forms the deep electron traps (bottom); the canonical 7,12-quinone and the 5,12-diketone are labelled.

Extending the screening across all six chemical classes resolves the map into six distinct electronic regimes (Table 1 and Figure 5), ranging from deep and shallow hole traps, through a “dual” trap and a gap-widening (i.e., locally dearomatised) regime, to mild and strong electron traps. Two consequences follow. First, a simple binary hole/electron classification is insufficient for the full landscape: the end-group epoxide can act simultaneously as a shallow hole trap and a deep electron trap, whereas the endoperoxides act as neither and merely widen the local gap. Second, and in contrast, the two stable end-members of the oxidation sequence—the hydroxyl and the quinone—obey the clean dichotomy of Section 3.2. Hence, even though the intermediate landscape is considerably richer, the dichotomy of the stable products remains a reliable and intuitive predictor.

It should be noted here that the open-shell radical intermediates (i.e., the alkoxyl/carbonyl and peroxyl species) are included in the map for completeness but are treated separately: their frontier level is singly occupied (SOMO) and is therefore not directly comparable, on the same quantitative axis, with the closed-shell HOMO/LUMO of the stable species. As a result, they are not employed to assign the measured signal.

### 3.5. Experimental Observation: A Hole Trap Resolved by DLTFS

**Figure 6:**
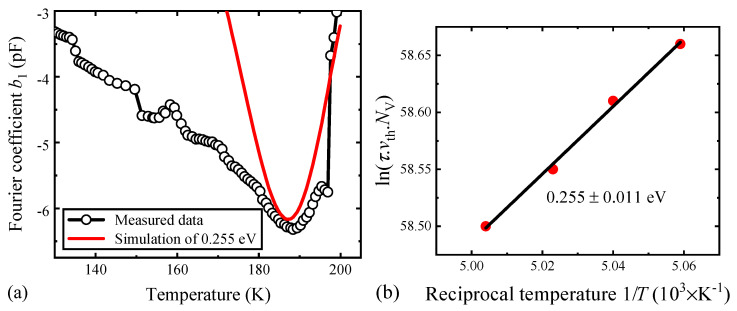
Experimental determination of the hole-trap activation energy by DLTFS. (**a**) DLTFS spectrum: the first Fourier sine coefficient $b_{1}$ as a function of temperature (130–205 K), showing the emission peak of the trap for the applied rate window. (**b**) The corresponding Arrhenius plot, $\ln(\tau \;v_{\mathrm{th}}\;N_{V})$ versus 1/T, from whose slope the activation energy $E_{a}=0.255\pm 0.011$ eV is obtained.

Deep-level transient Fourier spectroscopy on the DNTT transistors resolves a single hole trap. Its Arrhenius analysis (Figure 6) yields an activation energy $E_{a}=0.255\pm 0.011$ eV. It should be noted here that $E_{a}$ is obtained from the emission time constant $\tau_{e}$ (i.e., the time constant of the transient), and it is therefore independent of the amplitude-to-concentration conversion discussed in the Supplementary Materials.

### 3.6. A Defect-Level Rationalisation of the p/n Ambient-Stability Asymmetry

Collecting the whole map by trap polarity provides a compact, defect-level account of the well-known experimental observation that *p*-type acene semiconductors tolerate air far better than their *n*-type counterparts. On the hole side, the products of hydroxylation populate a band of shallow-to-moderate traps, with $\Delta E_{\mathrm{HOMO}}$ varying in a wide range from about +0.04 to +0.36 eV, which would slow down but not destroy hole transport. On the electron side, in contrast, the oxidation products cluster as deep electron traps: four independent carbonyl species—the canonical 7,12-*para*-quinone together with the 8,11- and 8,9-quinones and the 5,12-diketone—span $\Delta E_{\mathrm{LUMO}}$ from about −1.29 to −1.56 eV, and they are joined by the epoxides (−0.27 to −0.43 eV) and the terminal peroxyls (−0.43 to −0.48 eV); such levels would be catastrophic for electron transport. It should be noted here that this depth is a general feature of quinone and diketone formation and not an artefact of a single carbonyl geometry, since it persists across *para* and *ortho* regiochemistry and across inner- and outer-ring positions. In other words, the atmospheric oxidation of an acene generates predominantly shallow hole traps but deep electron traps so that hole (*p*-type) transport survives while electron (*n*-type) transport is essentially destroyed. It should be noted here that this is a statement about the oxidation landscape—i.e., about which species can form—and is decoupled from any particular device or exposure history; hence, it constitutes the strongest, experiment-independent result of the present map.

Against this landscape, the measured hole trap of about 0.255 eV dwells within the band that the sign rule predicts for hydroxylation and early oxidation. This is consistent with a photo-oxidative hydroxyl origin as one plausible assignment; however—owing to the semi-empirical nature of the proxy, the density of candidate states within this window, and the absence of an independent chemical or structural characterisation of the film—it cannot be regarded as a unique identification, and structural or contact-related origins cannot be excluded. We may therefore conclude that the map constrains the polarity and the plausible chemistry of the trap rather than its precise microscopic identity.

## 4. Discussion

### 4.1. Position of the Sign Rule and the p/n Asymmetry

The notion that a substituent shifts the frontier levels of a conjugated molecule according to its donating or accepting character is, in itself, elementary. What the present screening adds is the systematic application of this notion across an entire family of atmospheric oxidation products of a single semiconductor so that the trap polarity may be read directly from the sign of the frontier-level shift. Previous computational studies of traps in organic semiconductors have commonly addressed individual candidate species in isolation [14,15]; here, in contrast, the value dwells in the continuity and the breadth of the map, which turns a case-by-case assignment into a single organising rule.

The same breadth underlies the second and, arguably, the more general result. The markedly better air stability of *p*-type organic semiconductors relative to their *n*-type counterparts is well documented experimentally [7,8], and it has usually been attributed to oxidation and humidity in rather general terms. The present map provides, to our knowledge, the first defect-level rationalisation of this asymmetry: atmospheric oxidation generates predominantly shallow hole traps but deep electron traps so that hole transport is only mildly perturbed, whereas electron transport would be destroyed. It should be noted here that this account is decoupled from any particular device—being a statement about which species can form and where their frontier levels dwell—and it constitutes, for this reason, the most robust and experiment-independent result of the present study.

A further, independent line of support emerges when the map is confronted with the thermodynamic criterion for the ambient stability of *n*-type transport. De Leeuw and co-workers established that a reduced, electron-carrying state is stable against water and oxygen only if the electron affinity is sufficiently large—in practice, an electron affinity of roughly 3.5–4 eV, i.e., a LUMO near −4 eV—a threshold that later underpinned the deep-LUMO design rules for air-stable *n*-type semiconductors [27,28]. Notably, the oxidation-derived quinones of the present map fall within this window: added to the experimental pristine LUMO of DNTT (−2.44 eV [6]), the density-functional shifts of −1.25 to −1.86 eV (Section 4.4) place their absolute LUMO at about −3.7 to −4.3 eV; the semi-empirical shifts give essentially the same range (−3.7 to −4.0 eV) so that this placement does not depend on the level of the theory. Two consequences follow. First, these quinone states are deep enough to bind an electron stably in air so that they behave as permanent, thermodynamically robust electron traps rather than as reactive transients—consistent with their thermal silence in the DLTFS window. Second, and more broadly, the very deep LUMO that is deliberately engineered to obtain air-stable *n*-type transport is here produced spontaneously by oxidation of the acene but as isolated defects: the oxidative lowering of the LUMO that would (globally) be required for stable *n*-type conduction therefore (locally) destroys it, while perturbing hole transport only mildly so that the defect map supplies a microscopic basis for the macroscopic de Leeuw criterion. This absolute alignment, however, rests on adding a computed shift to the measured pristine level; owing to the semi-empirical, single-molecule nature of the screening, the agreement should be read as one of the right order rather than as an exact coincidence.

It should nevertheless be emphasised that the sign rule is a trend-level organising principle rather than a substitute for quantitative level positions; its strength dwells in predicting the polarity and the ordering of the traps rather than their absolute depths—a point to which we return below.

A remark on the relative likelihood of the mapped defects is in order, since not all of the identities are equally probable. The frontier reactivity index supplies an a priori ordering of the points of attack: the peri positions carry by far the largest HOMO population and are therefore the most susceptible to electrophilic oxidation (Figure 3) so that the peri hydroxyl and the peri-derived carbonyl/quinone—precisely the species that anchor the present analysis—are expected to dominate the early-stage defect population. The multiply-oxidised species (the bis-hydroxyls and the endoperoxides) and the high-locant single defects are best read as later-stage or less-probable products that delineate the boundaries of each electronic regime rather than as the most likely traps. It should be noted here that a quantitative formation-energy ranking would require explicit reference states for O_2_ and H_2_O together with a treatment of the reaction barriers, which lies outside the scope of the present frontier-orbital screening; the reactivity index is therefore used only as an ordinal proxy for susceptibility.

### 4.2. The Experimental Anchor and Why a Unique Identification Is Withheld

As shown in Section 3.6, the hole trap measured at about 0.255 eV dwells within the band that the sign rule predicts for hydroxylation and early oxidation, and it is, in this sense, fully consistent with a hydroxyl-related origin. It should be noted here, however, that consistency is deliberately not equated with identification, and this is due to three reasons.

First, this energy window is densely populated by chemically distinct species: the 12-hydroxyl at +0.181 eV, several bis-hydroxyl isomers between about +0.21 and +0.28 eV, and, numerically, the 12-carbonyl/alkoxyl radical at +0.255 eV. Hence a single activation energy cannot, on its own, single out one microscopic configuration. Second, the screening is semi-empirical, with an intrinsic scatter of the order of 0.1–0.3 eV; even a nominally exact coincidence therefore dwells well within the uncertainty of the method and carries no special weight. Third, and most importantly, the chemical composition of the degraded film was not characterised independently (e.g., by infrared or photoelectron spectroscopy), so the actual presence of hydroxyl groups is inferred rather than verified.

It should be emphasised that the apparent exact match of the 12-carbonyl radical is not employed here as an argument. Its frontier level is singly occupied (i.e., a SOMO) and is thus not directly comparable with the closed-shell levels, and the radical is, in any case, a transient intermediate rather than a stable trapping species. We therefore deliberately refrain from resting any identification on this coincidence.

Furthermore, the exposure history of the devices was short—the transistors were first measured about one day after fabrication and were subsequently held under vacuum—so that the extent of oxidation was necessarily limited, and origins other than hydroxylation cannot be excluded. In particular, the disruption of π–π stacking at grain boundaries [10], physisorbed oxygen or water acting as acceptors [7,9], and the penetration of silver from the evaporated top contacts all remain plausible contributors to a deep state of this energy. Indeed, a hole-trap feature at a comparable energy (about 0.25–0.35 eV above the mobility edge) has previously been resolved in DNTT and attributed to water-related states at the dielectric interface [7]. The chemical origin proposed here is therefore offered as a complementary—and, to our knowledge, previously unexplored—possibility and not as a replacement for these physical mechanisms. It should be noted here, moreover, that covalent oxidation defects are expected only in dilute concentrations, at or below the sensitivity of the composition-sensitive probes (XPS, FTIR, SIMS) that would be required to confirm them directly; their absence from earlier reports therefore reflects the difficulty of detecting such dilute chemical modifications—already electrically active as traps at concentrations far below the chemical detection floor—rather than their genuine absence. As a result, the map is best read as constraining the polarity and the plausible chemistry of the trap rather than as delivering its precise microscopic identity.

### 4.3. Optical Filling and the Absence of an Electron-Trap Signal

Because the traps were filled optically rather than by an electrical pulse, the green-light excitation (λ=520 nm, 2.38 eV) generates both holes and electrons within the DNTT layer so that—in principle—both the hole and the electron families of the map could be populated. Optical excitation of DNTT is indeed well documented to produce a photoresponse and to trap photo-generated carriers [9,29], the response being strongest near the absorption maximum at 460 nm [9] so that our 520 nm excitation lies on the low-energy tail. It should be noted here that the absence of any electron-related feature in the measured spectra is therefore not a filling artefact but rather a consequence of the detection stage.

The photo-generated species is initially a Frenkel exciton, the binding energy of which reaches a value of about 0.5 eV in acene-type materials [30]. Its dissociation into free carriers is driven by the high internal field established under reverse bias, together with charge separation at the semiconductor/dielectric interface and at defect sites. Interestingly, the deep oxidation-derived electron traps (i.e., the quinones and the 5,12-diketone, with $\Delta E_{\mathrm{LUMO}}$ reaching about −1.3 to −1.6 eV) constitute almost ideal electron sinks at which such a dissociation may proceed: the electron falls deep and remains captured, whereas the liberated hole is subsequently observed through its shallower hydroxyl-related trap. One and the same oxidation chemistry thus provides both a dissociation centre and the observed hole trap; this, however, should be regarded as a plausible mechanistic reading rather than an established claim.

The invisibility of the electron side then follows from a bundle of concurrent reasons. First, the deepest electron traps are thermally mute: at the measurement temperatures (130–205 K) a level lying 1.55 eV below the conduction-band minimum emits at a negligible rate and behaves as fixed charge rather than as a transient. Second, the structure is a *p*-type device measured under negative reverse bias, i.e., in hole accumulation, so that the capacitance transient is intrinsically governed by the exchange of holes with the valence band. Third, and in accordance with the two preceding points, the electron channel is doubly blocked: the effective electron mobility of DNTT is lower by orders of magnitude (the material being a strongly unipolar *p*-type semiconductor), and the silver source and drain present a large injection/extraction barrier to the shallow LUMO of DNTT: taking the DNTT HOMO and LUMO at about −5.44 and −2.44 eV, respectively [6], and the silver work function as 4.26 eV [31], the nominal (vacuum-level) electron injection barrier of about 1.8 eV substantially exceeds the hole barrier of about 1.2 eV. Hence, even a mid-depth electron trap that could in principle emit would find no communicating pathway to the electrodes.

It should be noted here that the resulting absence of an electron signal is thus an expected consequence of the measurement configuration and, crucially, does not constitute evidence concerning the electron side of the defect map. Where prior photoresponse studies of DNTT do resolve electron trapping, it is a reversible trapping of photo-generated electrons at the dielectric interface [9], distinct from the deep, covalent electron traps (the quinones) that the present map predicts. The *p*/*n* asymmetry of Section 3.6 therefore remains a computational prediction with which the present experiment is fully consistent (it resolves the hydroxyl-related hole trap) while being blind, by construction, to the electron-trapping species.

### 4.4. Methodological Standing: Validity and Limits of the Screening

The GFN2-xTB method employed here is a fast, broadly parametrised semi-empirical scheme, and its virtues and its limits should be stated plainly. Its absolute orbital energies are not reliable—the pristine HOMO of −9.76 eV is far removed from the reported ionisation energy of DNTT—so the method is used here not as a source of absolute levels but as a screening tool for the relative shifts and, above all, for their ordering. In other words, it is a powerful and inexpensive instrument for establishing trends, but it does not, on its own, deliver quantitative trap depths.

**Figure 7:**
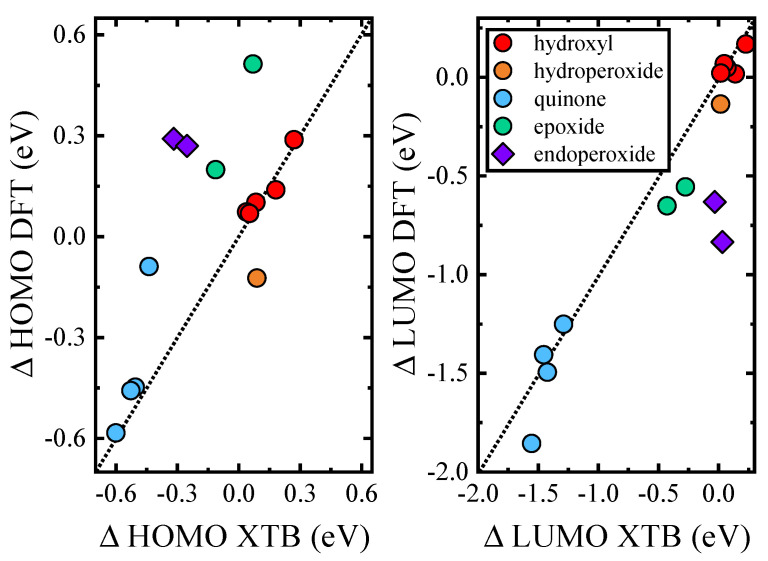
Validation of the semi-empirical screening against hybrid density-functional theory. Frontier-level shifts $\Delta E_{\mathrm{HOMO}}$ (**left**) and $\Delta E_{\mathrm{LUMO}}$ (**right**) relative to pristine DNTT, computed with GFN2-xTB (abscissa) and with B3LYP-D3BJ/def2-TZVP (ordinate) for fourteen closed-shell defects, coloured by chemical class; the dashed line is the 1:1 diagonal. The hole traps (hydroxyls) cluster at positive $\Delta E_{\mathrm{HOMO}}$ and the deep electron traps (quinones and the diketone) at strongly negative $\Delta E_{\mathrm{LUMO}}$ in both methods. The endoperoxides (diamonds) are the sole systematic exception (see text).

To test directly whether the sign rule and the ordering survive a higher level of theory, single-point hybrid density-functional calculations (B3LYP-D3BJ/def2-TZVP, with the RIJCOSX approximation) were carried out on the GFN2-xTB geometries of fourteen representative defects spanning all closed-shell classes—hydroxyls, a hydroperoxide, quinones, a diketone, epoxides and endoperoxides (Figure 7 and Table 2). At this level the pristine frontier levels are realistic (HOMO −5.25 eV, gap 3.12 eV, against the experimental −5.44 eV and ≈3.0 eV), and, more importantly, the polarity classification is reproduced throughout: every hydroxyl remains a hole trap ($\Delta E_{\mathrm{HOMO}}>0$ for all five), and every quinone together with the diketone remains a deep electron trap ($\Delta E_{\mathrm{LUMO}}$ between −1.25 and −1.86 eV). The two frameworks correlate closely—for $\Delta E_{\mathrm{LUMO}}$ across the twelve donor/acceptor defects, the Pearson coefficient is r=0.99—so that the sign rule, the ordering, and with them the *p*/*n* asymmetry are confirmed to be robust to the electronic-structure method. We verified, in addition, that the shifts are essentially unchanged between the def2-SVP and def2-TZVP basis sets, and they are therefore not an artefact of basis-set size.

One motif, however, exposes a genuine limit of the semi-empirical screening. For both endoperoxides the two methods disagree: GFN2-xTB classifies them as gap-wideners—a lowered HOMO with an essentially unshifted LUMO—whereas at the DFT level, the O–O bridge dearomatises the terminal ring so that the HOMO rises, the LUMO falls, and the gap in fact narrows. Because the two endoperoxides diverge in precisely the same way, the discrepancy is systematic rather than incidental, and it singles out the strained, dearomatised peroxide bridge as the one structural motif for which the semi-empirical frontier levels are not to be trusted. It should be noted here that this does not affect the conclusions of the present work, which rest on the hydroxyl and carbonyl/quinone channels; the endoperoxides are reported in full and are simply excluded from the quantitative correlation above.

A legitimate objection is that the single-molecule, gas-phase levels neglect the electronic polarisation of the surrounding crystal, which stabilises a localised charge. It should be noted here, however, that this polaronic term ($P^{+}\approx 0.05$–0.15 eV) is, to a good approximation, a state-independent rigid offset: it shifts the whole map downward but changes neither the sign of $\Delta E_{\mathrm{HOMO}/\mathrm{LUMO}}$ nor the ordering of the defects [24,25,26]. Hence, the two load-bearing conclusions of the present work—the trap polarity and the ordering of the levels—are invariant with respect to it. An explicit crystal-polarisation cell would refine the absolute depths further, but—as the density-functional validation below already demonstrates for the sign and the ordering—it would leave the polarity classification and the *p*/*n* asymmetry untouched; the absolute trap depths are therefore not pursued here.

Finally, it should be emphasised that the frontier reactivity index is used only in an ordinal sense. Because the minimal STO-3G basis and the Mulliken partitioning are known to be basis-set dependent, no quantitative weight is placed on its values; its sole role is to rank the sites a priori, that is, independently of the trap energetics. This independence is, in fact, crucial: it ensures that the most reactive site is identified from the intrinsic frontier density of the pristine molecule rather than being selected a posteriori because its energy happens to match the experiment, and it thereby removes the circularity that would otherwise threaten such an assignment.

### 4.5. Trap Concentration: A Cautionary Note

A word of caution is in order regarding the trap concentration which, unlike the activation energy, is not measured directly but is obtained by scaling the transient amplitude, $N_{T}=2N_{S}\left(\Delta C/C_{R}\right)$, by the shallow (doping) concentration $N_{S}$. For the present devices, the extraction returns an $N_{S}$ of about $3\times 10^{25}$ cm^−3^, which exceeds the molecular density of DNTT (approximately $2.7\times 10^{21}$ cm^−3^) by several orders of magnitude. It should be noted here that one cannot have more dopants than molecules; the extracted concentration is therefore an artefact rather than a physical value. The reason is simple: the standard analysis assumes a bulk Schottky junction with a bias-modulated depletion width, whereas the ≈50 nm, nearly intrinsic film is fully depleted so that its capacitance is geometric (bias-independent) and the extraction of $N_{S}$—and hence of $N_{T}$—breaks down. More generally, in a field-effect transistor, the charge is transported within a thin accumulation layer at the semiconductor/dielectric interface so that transfer-curve analyses probe essentially that interface and naturally return an areal trap density rather than a bulk one; the areal densities reported for DNTT devices, of the order of $10^{10}$–$10^{11}$ cm^−2^ [7,9], are consistent with this picture. As this behaviour is returned as a matter of course by commonly used commercial DLTS systems, the reported concentration should not be taken at face value; the detailed analysis is given in the Supplementary Materials. Crucially, the activation energy $E_{a}$ is obtained from the emission time constant $\tau_{e}$ and not from the amplitude, so that it—and with it the entire defect map—is unaffected by this limitation.

### 4.6. Implications

The practical implication of the present work is that the map constitutes a roadmap linking oxidation chemistry to trap polarity, which can be used to assign the electronic character of a trap once it has been measured: given an activation energy together with its sign, the map indicates the class of degradation product to which the level may correspond, without a dedicated calculation for each new case. Since the underlying rule—the sign of the frontier shift, set by the donor or acceptor nature of the oxygen functionalisation—is chemical rather than specific to DNTT, the same approach should be transferable to other acene semiconductors. In particular, the rule is expected to carry over to the widely used side-chain- and core-modified DNTT derivatives—such as the dialkyl C_*n*_-DNTTs and the diphenyl variant—since the alkyl or aryl groups govern solubility and film packing but leave the conjugated core, on which the oxygen functionalisation acts, essentially intact; the heavier chalcogen (seleno) analogues likewise preserve the donor/acceptor logic of the sign rule, even though their absolute frontier levels are shifted. The polarity classification should therefore transfer to these technologically important materials, although the precise trap depths would have to be recomputed in each case. Hence, the screening offers an inexpensive and reusable reference for interpreting trap spectra in atmospherically degraded organic devices.

## 5. Conclusions

We have screened 39 oxygen- and hydroxyl-related defect identities of DNTT with the semi-empirical GFN2-xTB method and shown that the sign of the frontier-level shift governs the trap polarity: a π-donating hydroxyl raises the HOMO and yields a hole trap, whereas a π-accepting carbonyl or quinone lowers the frontier levels and yields a deep electron trap. The main result is that this rule provides, to our knowledge, the first defect-level rationalisation of the *p*/*n* ambient-stability asymmetry of acene semiconductors: hydroxylation generates only shallow-to-moderate hole traps ($\Delta E_{\mathrm{HOMO}}$ of about +0.04 to +0.36 eV), which slow but do not destroy hole transport, whereas oxidation to quinones and diketones generates deep electron traps ($\Delta E_{\mathrm{LUMO}}$ of about −1.3 to −1.6 eV across four independent carbonyl species), which would be catastrophic for electron transport. These sign and ordering trends are reproduced by hybrid density-functional theory (B3LYP/def2-TZVP) so that the classification is robust to the level of theory. Against this map, a hole trap of 0.255 eV measured by DLTFS is consistent with a hydroxyl-related origin, although—owing to the semi-empirical proxy and to the absence of an independent chemical characterisation—a unique microscopic assignment is not claimed. The map thus constitutes a reusable roadmap linking oxidation chemistry to trap polarity and, since the underlying rule is chemical rather than specific to DNTT, it should be transferable to other acene semiconductors. It should be noted here that the present trends could be sharpened by an ageing series together with spectroscopic identification (FTIR, XPS, or EPR) of the degradation products, and the absolute trap depths by a ΔSCF treatment of the radicals and an explicit crystal-polarisation treatment, all of which lie beyond the scope of the present screening.

## Figures and Tables

**Table 1:** Representative member of each of the six electronic regimes resolved by the screening (closed-shell species). Values are frontier-level shifts relative to pristine DNTT, in eV. The complete 39-identity table is given in the Supplementary Materials, and representative chemical structures of the classes are shown in Figure S2.

Regime	$\Delta E_{\mathrm{HOMO}}$	$\Delta E_{\mathrm{LUMO}}$	Representative Defect
Deep hole trap	+0.36	+0.27	5,12-(OH)_2_
Shallow hole trap	+0.05	+0.02	10-OH
Dual trap	+0.07	−0.43	9,10-epoxide
Gap-widener	−0.32	+0.03	7,12-endoperoxide
Mild electron trap	−0.11	−0.27	11a,12-epoxide
Strong electron trap	−0.51	−1.29	7,12-*para*-quinone (canonical)

Open-shell radical intermediates form an additional (SOMO-based) group that is reported separately and not placed on this axis.

**Table 2:** Representative comparison of the frontier-level shifts (relative to pristine DNTT, in eV) from the GFN2-xTB screening and from single-point B3LYP-D3BJ/def2-TZVP. The complete set of fourteen defects is shown in Figure 7.

Defect	$\Delta E_{\mathrm{HOMO}}$	$\Delta E_{\mathrm{HOMO}}$	$\Delta E_{\mathrm{LUMO}}$	$\Delta E_{\mathrm{LUMO}}$	Character
	**(xTB)**	**(DFT)**	**(xTB)**	**(DFT)**	
12-OH	+0.18	+0.14	+0.14	+0.02	hole trap
4,12-(OH)_2_	+0.27	+0.29	+0.23	+0.17	hole trap
12-OOH	+0.09	−0.12	+0.02	−0.14	near-neutral
7,12-quinone	−0.51	−0.45	−1.29	−1.25	deep *e*-trap
5,12-diketone	−0.44	−0.09	−1.56	−1.86	deep *e*-trap
8,9-quinone	−0.60	−0.58	−1.42	−1.49	deep *e*-trap
9,10-epoxide	+0.07	+0.51	−0.43	−0.65	dual
7,12-endoperoxide	−0.32	+0.29	+0.03	−0.83	xTB fails

## Data Availability

The raw data supporting the conclusions of this article will be made available by the authors on request; the key data are included in the Supplementary Materials.

## Supplementary Materials

The following supporting information can be downloaded at https://www.mdpi.com/article/10.3390/ma19153333/s1, Section S1: Trap-concentration analysis: capacitance–voltage of the DNTT MIS structure. Figure S1: (a) Capacitance–voltage and (b) $1/C^{2}$ characteristics of the DNTT MIS structure. The accumulation plateau pins the capacitance to $C_{\mathrm{ox}}$, so the $1/C^{2}$ branch fitted by the system (red line) is nearly flat; the resulting Mott–Schottky extraction yields the unphysical $N_{S}$, $U_{D}$ and $\varphi_{B}$ quoted in the text. Section S2: Complete defect map. Section S3: Additivity of bis-hydroxyl shifts. Table S1: Complete map of the 39 computed oxygen- and hydroxyl-related defect identities of DNTT (GFN2-xTB). HOMO, LUMO and their shifts ΔHOMO, ΔLUMO relative to pristine DNTT, and the gap, are in eV; the dipole moment (dip) is in D. Open-shell radicals (conf = open) are singly occupied: for these the ΔHOMO column reports ΔSOMO, which is not directly comparable with the closed-shell values, and the LUMO and gap are not tabulated. Figure S2: Representative chemical structures of the defect classes discussed in the main text: (a) pristine DNTT; (b) the peri hydroxyl (12-OH); (c) the peri hydroperoxide (12-OOH); (d) the 7,12-*para*-quinone; (e) the 5,12-diketone; (f) the 9,10-epoxide; and (g) the 7,12-endoperoxide. The identifiers match those used in Table 1 and Figure 5 of the main text and the data of Table S1. Section S4: The ortho-catechol sub-additive outlier. Table S2: Additivity of the bis-hydroxyl HOMO shifts (eV): the additive prediction from the two constituent mono-hydroxyls, the computed value, and the relative deviation. Section S5: Thienothiophene-bridge delocalisation. Section S6: α-Oxygen Mulliken charges. Table S3: α-oxygen Mulliken charge q(αO) (in *e*) of the peroxyl and hydroperoxide defects at the six ring positions of DNTT.
