# Supplementary material for: Trap Polarity and the p/n Asymmetry in Oxidised DNTT: A Frontier-Shift Rule
Source: Materials (Basel). 2026 Aug 5;19(15):3333. doi: 10.3390/ma19153333 (PMC13468534; doi:10.3390/ma19153333)
Supplement: Supplementary file 1 [file materials-19-03333-s001.zip › materials-4455586-supplementary.pdf]

Supplementary Materials for: Trap Polarity and the p/n Asymmetry in Oxidised DNTT: A Frontier-Shift Rule

M. Matuš, T. Vincze, M. Hanic, L. Stuchlikova, M. Weis

S1 Trap-concentration analysis: capacitance–voltage of the DNTT MIS structure

The trap concentration returned by the DLTS system is not measured directly; it is obtained by scaling the transient amplitude,

$$N_T = 2\,N_S\,\frac{\Delta C}{C_R}, \tag{S1}$$

by the shallow (doping) concentration N_S, which the system extracts from the capacitance–voltage (C–V) characteristic. For the present devices this route returns a physically impossible value, for the reasons set out below. Because the extraction rests entirely on N_S, the activation energy—obtained from the emission time constant τ_e (Arrhenius analysis), not from the amplitude—is unaffected by any of the following.

A categorical model error. The measured device is a metal–insulator–semiconductor (MIS) capacitor (highly doped Si gate / 100 nm thermal SiO_2 / DNTT, top Ag contacts), but the system applied a Schottky (Mott–Schottky) C–V model, which is the root cause. The recorded capacitance (Figure S1) returns $N_S = N_{SP} = 3.09 \times 10^{25}$ cm^{-3}, a diffusion voltage $U_D = -103.9$ V, a barrier $\varphi_B = 103.1$ eV and a fit correlation of only 0.943. These values are unphysical: N_S exceeds the molecular density of DNTT ($\approx 2.7\times 10^{21}$ cm^{-3}) by about four orders of magnitude—one cannot have more dopants than molecules—while φ_B merely mirrors $|U_D|$ and the correlation of 0.943 confirms that the $1/C^2$ characteristic is not linear.

Why the extraction diverges. In a MIS structure the accumulation side is pinned to the oxide capacitance C_{ox}, so that $1/C^2$ versus bias is nearly flat (indeed slightly decreasing) over the branch on which the regression lies, whereas the Mott–Schottky analysis requires a linear *increase*. The extracted slope tends to zero (or becomes negative); since $N_S \propto 1/\text{slope}$, a gigantic N_S results and the intercept extrapolates to the spurious $U_D \approx -104$ V.

A secondary area error. The nominal area $F = 8 \times 10^{-3}$ cm^2 (0.8 mm^2) underestimates the true device area. From the accumulation capacitance ($C \to C_{\text{ox}} \approx 700$ pF), the effective area is

$$A_{\text{eff}} = \frac{C_{\text{ox}}\,d_{\text{ox}}}{\varepsilon_0\varepsilon_r} = \frac{700\text{ pF} \times 100\text{ nm}}{\varepsilon_0 \times 3.9} \approx 2.0 \times 10^{-2}\text{ cm}^2, \tag{S2}$$

about 2.5 times the nominal value. As $N_S \propto 1/F^2$, this inflates N_S by a further factor of $(2.0/0.8)^2 \approx 6.3$. It should be stressed, however, that correcting the area alone does not resolve the discrepancy: even then N_S would remain of the order of 5×10^{24} cm^{-3}, still far above the molecular density. The dominant error is the model, not the area.

Full depletion of the thin film. At the few-volt bias used, the C–V is saturated, indicating that the $\approx$ 50 nm, nearly intrinsic film is fully depleted. The capacitance is then geometric ($C = \varepsilon A/d$, bias-independent), so the depletion width is pinned to the film thickness and cannot be modulated. The bulk assumption on which Equation (S1) rests—a bias-modulated depletion width in a semi-infinite semiconductor—therefore fails, and the extraction of N_S (and hence of N_T) is invalid.

Consequence. In a fully depleted film a *volume* trap concentration is not a well-defined quantity; only an areal trap density N_{ss} (cm^{-2}) is meaningful, subject to the hard cap $N < 2.7 \times 10^{21}$ cm^{-3}. The concentration of the order of 10^{23} cm^{-3} returned by the standard analysis should therefore be regarded as an artefact of applying a bulk Schottky model to a thin-film MIS structure, and not taken at face value. A physically meaningful extraction would require subtracting the series oxide capacitance ($1/C = 1/C_{\text{ox}} + 1/C_{\text{HL}}$) and restricting the Mott–Schottky fit to a genuinely linear depletion branch, using A_{eff} rather than the nominal area.

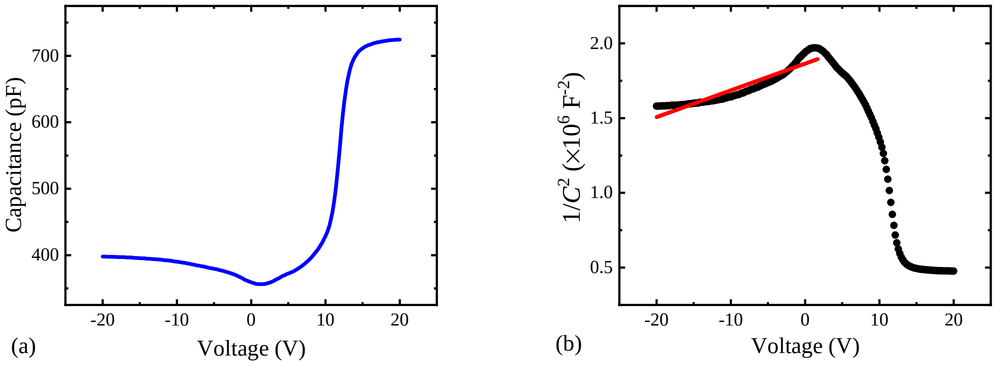

Figure S1: (a) Capacitance–voltage and (b) $1/C^2$ characteristics of the DNTT MIS structure. The accumulation plateau pins the capacitance to C_{ox}, so the $1/C^2$ branch fitted by the system (red line) is nearly flat; the resulting Mott–Schottky extraction yields the unphysical N_S, U_D and φ_B quoted in the text.

S2 Complete defect map

Figure S2 shows representative chemical structures for the closed-shell defect classes; the complete numerical map follows in Table S1.

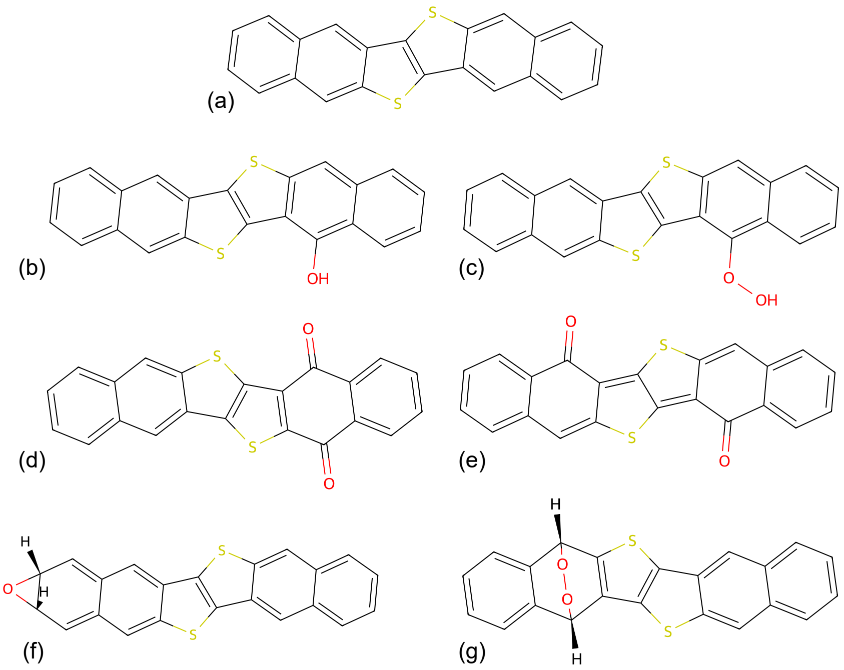

Figure S2: Representative chemical structures of the defect classes discussed in the main text: (**a**) pristine DNTT; (**b**) the peri hydroxyl (12-OH); (**c**) the peri hydroperoxide (12-OOH); (**d**) the 7,12-*para*-quinone; (**e**) the 5,12-diketone; (**f**) the 9,10-epoxide; and (**g**) the 7,12-endoperoxide. The identifiers match those used in Table 1 and Figure 5 of the main text and the data of Table S1.

S3 Additivity of bis-hydroxyl shifts

For the bis-hydroxyls the HOMO shift is, to a good approximation, the sum of the shifts of the two constituent mono-hydroxyls,

$$\Delta E_{\mathrm{HOMO}}(\mathrm{A},\mathrm{B}) \approx \Delta E_{\mathrm{HOMO}}(\mathrm{A}) + \Delta E_{\mathrm{HOMO}}(\mathrm{B}). \tag{S3}$$

Table S2 compares this additive prediction with the computed value for the five non-catechol bis-hydroxyls (positions related by the molecular inversion symmetry, e.g. 4 and 11, are treated as equivalent); the agreement is within a few percent throughout. This near-additivity reflects the essentially independent, local nature of each hydroxyl perturbation and underlies the linear reactivity–depth relation of the main text. The single exception, the ortho-catechol, is discussed in Section S4.

S4 The ortho-catechol sub-additive outlier

The 8,9-$(\mathrm{OH})_2$ defect, in which the two hydroxyls occupy adjacent (*ortho*) positions, departs strongly from additivity: its computed shift of +0.037 eV lies far below the additive prediction of +0.121 eV, a reduction of about 69%. The origin is an intramolecular O–H$\cdots$O hydrogen bond between the neighbouring hydroxyls, which ties up one oxygen lone pair and thereby suppresses its π-donation into the ring. The pair therefore acts as a much weaker donor than two independent hydroxyls, and the ortho-catechol emerges as the shallowest of the bis-hydroxyl hole traps.

Table S1: Complete map of the 39 computed oxygen- and hydroxyl-related defect identities of DNTT (GFN2-xTB). HOMO, LUMO and their shifts ΔHOMO, ΔLUMO relative to pristine DNTT, and the gap, are in eV; the dipole moment (dip) is in D. Open-shell radicals (conf = open) are singly occupied: for these the ΔHOMO column reports ΔSOMO, which is not directly comparable with the closed-shell values, and the LUMO and gap are not tabulated.

Defect	conf	HOMO	LUMO	ΔHOMO	ΔLUMO	gap	dip
Pristine DNTT	closed	−9.7598	−7.7751	0.000	0.000	1.985	0.00
Class A — Hydroxyls							
10-OH	closed	−9.7077	−7.7547	+0.052	+0.020	1.953	1.73
9-OH	closed	−9.7222	−7.7276	+0.038	+0.048	1.995	1.23
8-OH	closed	−9.6770	−7.7046	+0.083	+0.071	1.972	1.50
11-OH	closed	−9.6655	−7.6824	+0.094	+0.093	1.983	—
7-OH	closed	−9.6214	−7.6943	+0.138	+0.081	1.927	1.52
12-OH	closed	−9.5789	−7.6345	+0.181	+0.141	1.944	—
8,9-$(OH)_2$ (catechol)	closed	−9.7225	−7.7943	+0.037	−0.019	1.928	2.27
9,12-$(OH)_2$	closed	−9.5497	−7.5851	+0.210	+0.190	1.965	1.89
7,11-$(OH)_2$	closed	−9.5304	−7.6031	+0.229	+0.172	1.927	3.05
4,12-$(OH)_2$ (cross)	closed	−9.4907	−7.5463	+0.269	+0.229	1.944	0.45
11,12-$(OH)_2$	closed	−9.4806	−7.5245	+0.279	+0.251	1.956	3.85
7,12-$(OH)_2$	closed	−9.4388	−7.5840	+0.321	+0.191	1.855	3.40
5,12-$(OH)_2$	closed	−9.4033	−7.5026	+0.357	+0.272	1.901	0.00
Class B — Alkoxyl/carbonyl radicals (open-shell, SOMO)							
12=$O^{\bullet}$	open	−9.5052	—	+0.255	—	—	3.87
7=$O^{\bullet}$	open	−9.5550	—	+0.205	—	—	4.40
11=$O^{\bullet}$	open	−9.6460	—	+0.114	—	—	5.76
8=$O^{\bullet}$	open	−9.6898	—	+0.070	—	—	5.91
9=$O^{\bullet}$	open	−9.7644	—	−0.005	—	—	9.00
10=$O^{\bullet}$	open	−9.8168	—	−0.057	—	—	8.27
Class C — Peroxyl radicals (open-shell)							
12-O-$O^{\bullet}$	open	−9.7077	−7.9261	+0.052	−0.151	1.782	4.91
7-O-$O^{\bullet}$	open	−9.7417	−8.1111	+0.018	−0.336	1.630	6.56
11-O-$O^{\bullet}$	open	−9.7473	−8.0805	+0.013	−0.305	1.667	6.20
8-O-$O^{\bullet}$	open	−9.8978	−8.2023	−0.138	−0.427	1.695	8.11
9-O-$O^{\bullet}$	open	−9.8966	−8.2522	−0.137	−0.477	1.644	10.17
10-O-$O^{\bullet}$	open	−9.9511	−8.2369	−0.191	−0.462	1.714	8.82
Class D — Hydroperoxides							
12-O-OH	closed	−9.6722	−7.7583	+0.088	+0.017	1.914	1.01
7-O-OH	closed	−9.7806	−7.8339	−0.021	−0.059	1.947	1.12
11-O-OH	closed	−9.7311	−7.7738	+0.029	+0.001	1.957	0.95
8-O-OH	closed	−9.8321	−7.8546	−0.072	−0.079	1.977	2.35
9-O-OH	closed	−9.8491	−7.9211	−0.089	−0.146	1.928	3.35
10-O-OH	closed	−9.9043	−7.9440	−0.144	−0.169	1.960	3.45
Class E — Quinones and diketone							
5,12-diketone (*trans*)	closed	−10.1980	−9.3300	−0.438	−1.555	0.867	0.00
7,12-quinone (*para*)	closed	−10.2660	−9.0640	−0.506	−1.289	1.202	1.67
8,11-quinone (*para*)	closed	−10.2870	−9.2300	−0.527	−1.455	1.057	4.49
8,9-quinone (*ortho*)	closed	−10.3600	−9.1990	−0.600	−1.424	1.161	9.09
Class F — Cyclic (endoperoxides, epoxides)							
7,12-endoperoxide	closed	−10.0790	−7.7432	−0.319	+0.032	2.336	3.68
8,11-endoperoxide	closed	−10.0135	−7.8065	−0.254	−0.031	2.207	3.72
11a,12-epoxide	closed	−9.8727	−8.0493	−0.113	−0.274	1.823	2.23
9,10-epoxide	closed	−9.6903	−8.2030	+0.069	−0.428	1.487	2.24

Table S2: Additivity of the bis-hydroxyl HOMO shifts (eV): the additive prediction from the two constituent mono-hydroxyls, the computed value, and the relative deviation.

Bis-hydroxyl	Constituents	Additive	Computed	Dev.
9,12-$(OH)_2$	9-OH + 12-OH	+0.219	+0.210	−4.1%
7,11-$(OH)_2$	7-OH + 11-OH	+0.232	+0.229	−1.3%
4,12-$(OH)_2$	4-OH + 12-OH	+0.275	+0.269	−2.2%
11,12-$(OH)_2$	11-OH + 12-OH	+0.275	+0.279	+1.5%
7,12-$(OH)_2$	7-OH + 12-OH	+0.319	+0.321	+0.6%

S5 Thienothiophene-bridge delocalisation

The two naphthalene wings of DNTT are not electronically isolated: the frontier orbital delocalises across the central thienothiophene bridge. This is shown by comparing two bis-hydroxyls with the *same* additive prediction: 11,12-$(OH)_2$, with both hydroxyls on the same wing, and 4,12-$(OH)_2$, with one hydroxyl on each wing (positions 4 and 11 being related by the molecular inversion symmetry). Their computed shifts, +0.279 and +0.269 eV, differ by only about 10 meV, so that the cross-wing defect retains roughly 96% of the same-wing shift. In other words, the thienothiophene bridge transmits the HOMO delocalisation between the wings almost without loss, so that a hydroxyl on one wing is felt, electronically, on the other.

S6 α-Oxygen Mulliken charges

The progressive loss of π-donor strength across the three single-oxygen species at a given site, invoked in Section 3.3 of the main text, is quantified by the Mulliken charge on the oxygen bonded to the ring. Table S3 lists the α-oxygen charge $q(\alpha\mathrm{O})$ for the peroxyl and hydroperoxide families at the six ring positions. For the closed-shell hydroxyls the single oxygen carries $q(\mathrm{O}) \approx -0.38$ (nearly position-independent; the computed mono-hydroxyls range from −0.381 to −0.390), placing them at the strong-donor end of the sequence hydroxyl (≈ -0.38) $\rightarrow$ hydroperoxide (≈ -0.17) $\rightarrow$ peroxyl (≈ -0.04).

Table S3: α-oxygen Mulliken charge $q(\alpha\mathrm{O})$ (in e) of the peroxyl and hydroperoxide defects at the six ring positions of DNTT.

Position	Hydroperoxide $q(\alpha\mathrm{O})$	Peroxyl $q(\alpha\mathrm{O})$
12 (peri)	−0.167	−0.041
7	−0.184	−0.036
11	−0.182	−0.035
8	−0.148	−0.036
9	−0.152	−0.036
10	−0.149	−0.034
